# Production Yield and Meat Quality Characteristics of Six Pork Head Cuts from Commercial Crossbred Pigs

**DOI:** 10.3390/foods15193367

**Published:** 2026-09-22

**Authors:** Kyung Jo, Van Ba Hoa, Suk Han Jung, Dong-Gyun Kim, Sun Moon Kang

**Affiliations:** Animal Resources Food Tech Division, National Institute of Animal Science, Rural Development Administration, 1500 Kongjwipatjwi-ro, Iseo-myeon, Wanju-gun 55365, Jeonbuk State, Republic of Korea; kung6321@korea.kr (K.J.); hoavanba@korea.kr (V.B.H.); tnr12gks12@naver.com (S.H.J.); kdg6589@korea.kr (D.-G.K.)

**Keywords:** pork head, pork cut, production yield, meat quality, slaughter weight

## Abstract

Although pork head meat is an edible resource, information on the quality of individual cuts remains limited. In this study, we investigated the effects of slaughter weight on the yields of pork heads and individual pork head cuts, and evaluated the physicochemical properties of individual cuts using 150 commercial crossbred pigs. The quality properties of occipital, cheek, nasal, temporal, jowl, and sublingual meat obtained from pigs in the 110–119 kg slaughter weight group were analyzed. As slaughter weight increased, the absolute yields of pork head and individual head cuts increased, whereas their relative yields did not differ significantly among the slaughter weight groups. Individual pork head cuts also exhibited different quality properties. Nasal and sublingual meats had high pH values, a* values, total myoglobin content, and polyunsaturated fatty acid (PUFA) proportions, whereas jowl meat had high fat content, L* values, low total myoglobin content, high monounsaturated fatty acid (MUFA) proportion, and low PUFA proportion. These results suggest that individual pork head cuts have distinct properties and provide baseline information for quality evaluation and utilization.

## 1. Introduction

Pork is one of the major animal-source foods consumed worldwide. In 2024, pork consumption was 20.31 kg/capita in OECD countries, and in particular 30.1 kg/capita in Korea [1,2]. Pig slaughter and processing generate various edible by-products and underutilized portions that contain valuable nutrients, including proteins, fats, and minerals [3,4]. From the perspective of sustainability in the meat industry, reducing the loss of edible by-products and underutilized portions and promoting their efficient utilization have become increasingly important [3]. However, the industrial utilization of these materials is frequently limited because studies on their yields and quality characteristics remain insufficient [5]. Therefore, a systematic evaluation of edible pork by-products is required to enhance their value and expand their potential applications.

Pork head meat is the edible part of the porcine head and contains 79% boneless meat and 21% tongue [6]. Boneless meat obtained from pork heads can be categorized into several cuts at different anatomical locations, including occipital, cheek, temporal, nasal, jowl, and sublingual meats. Previous studies have evaluated the use of pork head meat as a raw material in processed meat products such as frankfurters and hamburger patties [6,7]. However, studies on the yield and quality characteristics of individual pork head cuts remain limited compared to those of conventional pork cuts such as loin, belly, and leg.

The composition and quality of pork head meat may vary because it comprises cuts from different anatomical locations. These attributes are related to visual appearance, as well as to processing suitability, nutritional characteristics, and potential product applications. Slaughter weight also affects the absolute yield and carcass composition of conventional pork cuts. However, limited information is available regarding changes in the yields of pork heads and their individual cuts according to slaughter weight. Therefore, in this study, we investigated the changes in the yields of pork heads and six individual pork head cuts according to slaughter weight. The features of the six cuts were compared to characterize their quality traits.

## 2. Materials and Methods

### 2.1. Animals and Sample Collection

All the animal procedures were approved by the Institutional Animal Care and Use Committee of the National Institute of Animal Science (NIAS), Rural Development Administration, Republic of Korea (approval number: NIAS–2020-0434). Slaughter was conducted at the NIAS slaughter facility in accordance with the Enforcement Rule of the Livestock Products Sanity Control Act of Korea [8]. In total, 150 commercial crossbred pigs (Landrace × Yorkshire × Duroc) were obtained from multiple local commercial farms. The study population comprised 96 castrated males and 54 females. Pigs were classified into six slaughter weight groups, as presented in Table 1: (1) 80–89 kg (*n* = 10); (2) 90–99 kg (*n* = 25); (3) 100–109 kg (*n* = 35); (4) 110–119 kg (*n* = 62); (5) 120–135 kg (*n* = 8); (6) 190–225 kg (*n* = 10). Under the current pork marketing structure, pigs weighing 190–225 kg substantially exceed the typical finishing weight and are marketed as culled breeding sows; therefore, this group consisted exclusively of culled breeding sows. Following arrival at the slaughter facility, the pigs were subjected to 5–6 h of lairage before slaughter. Electrical stunning (220 V) was applied, followed by exsanguination by severing the carotid arteries, while the animals were suspended by the hind legs. Carcasses were scalded with steam at approximately 60 °C, followed by mechanical dehairing, and subsequently singed to remove residual hair. Heads were separated from the carcasses by cutting between the occipital bone and the first cervical vertebra, washed with potable water, and then chilled at 1 °C for 24 h. After weighing the heads, six pork head cuts–occipital, cheek, nasal, temporal, jowl, and sublingual meat –were prepared according to fabrication procedures typically used in commercial pork head-processing facilities (Figure 1). The individual pork head cuts are shown in Figure 2, and their yields were determined.

In the present study, “pork head” refers to the whole anatomical head structure separated from the carcass, whereas “pork head cuts” refers to the six individual edible cuts evaluated in this study.

Based on the average slaughter weight of market pigs in Korea (116 kg), the 110–119 kg group was selected for meat quality evaluation. In this group, 40 castrated males were randomly selected to minimize sex-related variation. Pork head cuts were obtained from these 40 pigs, resulting in 40 samples per cut. After all visible connective tissue and subcutaneous fat had been removed, the 40 samples of each cut were randomly allocated to four analysis sets of 10 samples each: (1) proximate composition, energy content, collagen content, and fatty acid profile; (2) total myoglobin content, pH, water-holding capacity (WHC), and CIE color values; (3) purge loss; and (4) cooking loss.

### 2.2. Reagents and Chemicals

Deionized water (DW) was prepared using a Milli-Q purification system (Millipore S.A.S, Molsheim, Alsace, France). Chloroform and methanol were sourced from J. T. Baker (Philipsberg, NJ, USA). All other reagents and chemicals were supplied by Sigma-Aldrich Co. LLC. (St. Louis, MO, USA).

### 2.3. Determination of Pork Head Cut Yield

The weight (kg) of each head cut was recorded using a digital balance (ML6002T/00, Mettler-Toledo GmbH, Greifensee, Zurich, Switzerland) with a precision of 0.01 g. Relative yield (%) was calculated based on the slaughter weight (kg) of each pig.

### 2.4. Proximate Composition and Collagen Content Analysis

Moisture, fat, protein, and collagen contents were analyzed with a near-infrared spectrophotometer (FoodScan™ 2, Foss Analytical A/S, Hillerød, Hovedstaden, Denmark) according to AOAC official methods 2007.04 [9]. Collagen content was determined using the manufacturer’s calibration program for meat products. The results were expressed as g of each component per 100 g of meat.

### 2.5. Energy Content Determination

Energy content was calculated by multiplying the fat (9.02) and protein (4.27) contents by the energy conversion factors recommended by the FAO [10]. The result was expressed as kcal per 100 g of meat.

### 2.6. pH Measurement

pH was measured as described by Suman et al. [11]. Ten g of ground meat was homogenized with 100 mL of DW at 968 g for 30 s using a Polytron device (PT-MR 2100, Kinematica AG, Littau, Luzern, Switzerland). The pH value of the homogenate was measured using a pH meter (SevenEasy pH 20K, Mettler-Toledo GmbH) equipped with an InLab^®^ Expert Pro-ISM probe (Mettler-Toledo GmbH). Before measuring, the pH meter was calibrated at room temperature (24–25 °C) using three standard buffer solutions (pH 7.00, 4.01, 9.21; Mettler-Toledo GmbH).

### 2.7. Water-Holding Capacity Determination

The WHC was determined as previously described [12] using a high-speed centrifuge. Briefly, ground meat (1 g) was weighed into a centrifuge tube and then centrifuged at 70,000× *g* for 30 min at 10 °C (Avanti JXN-26 Centrifuge, Beckman Coulter, Inc., Fullerton, CA, USA). The expressed juice collected at the bottom of the centrifuge tube was absorbed onto a filter paper (No. 2, Whatman PLC, Maidstone, Kent, UK) for 30 min. The results were expressed as the percentage (%) of water content retained after centrifugation relative to the initial water content of the meat.

### 2.8. Purge Loss Determination

The purge loss was determined as previously described [13]. The meat was vacuum-packaged and stored at 4 °C. On day 7 of storage, the surface exudate was removed using Whatman No. 2 filter paper prior to measuring the meat weight. The results were calculated as the percentage (%) of weight loss relative to the initial meat weight.

### 2.9. Cooking Loss Determination

The cooking loss was determined using the procedure described by Honikel [14]. The meat was placed in a low-density polyethylene (LDPE) zipper bag, and then heated in a water bath (WSB-45, Daihan Scientific Co., Ltd., Seoul, Republic of Korea) at 80 °C until the internal temperature reached 75 °C. During heating, the internal temperature was monitored using a digital thermometer (307 B; Tecpel Co., Ltd., Xindian, New Taipei, Taiwan). Before weighing, the cooked meat was cooled in ice water and held at 4 °C for 24 h. The results were calculated as the percentage (%) of weight loss relative to the initial meat weight.

### 2.10. Total Myoglobin Content Measurement

Total myoglobin content was measured as previously described [15]. Ground meat (5 g) was homogenized with 20 mL of ice-cold DW at 1296× *g* for 1 min using an Ultra-Turrax device (T25 Digital, Ika Werke GmbH & Co., Staufen, Baden-Württemberg, Germany) and centrifuged at 30,000× *g* for 30 min at 2 °C (Avanti J-E Centrifuge, Beckman Coulter, Inc., Fullerton, CA, USA). After filtering through two layers of gauze and a 0.45 μm syringe filter, the absorbance value of the supernatant was read at 525 nm using a UV/Visible spectrophotometer (UV-1280, Shimadzu Corp., Kyoto, Kansai, Japan). The result was calculated as a milligram of myoglobin per gram of meat using the molecular weight (16,110 Da) and the millimolar extinction coefficient (7.6 mM^−1^ cm^−1^) of myoglobin.

### 2.11. CIE Color Value Measurement

After wrapping with the LDPE film, the meat was allowed to bloom at 4 °C for 30 min. L* (lightness), a* (redness), b* (yellowness), C* (chroma = (a*^2^ + b*^2^)^0.5^), and h° (hue-angle = tan^−1(b*/a*)^) values [16] were measured using a chroma meter (CR-400, Konica Minolta, Inc., Osaka, Kansai, Japan) equipped with a data processor (DP-400, Konica Minolta, Inc.), containing a 2° standard observer, 8 mm aperture, and light source set at illuminant C. Prior to measuring, the chroma meter was calibrated with a white standard tile (CR-A43, Konica Minolta, Inc.; L* = 97.46, a* = 0.08, and b* = 1.81).

### 2.12. Fatty Acid Profile Analysis

According to a method previously described [17], lipids were extracted using a chloroform:methanol (2:1, *v*/*v*) solution, and the solvent was removed at 38 °C under a stream of nitrogen gas (MGS-2200, Eyela Tokyo Rikakikai Co., Ltd., Tokyo, Honshu, Japan). The dried lipid was transesterified to fatty acid methyl ester (FAME) at 80 °C using 0.5 N NaOH (in methanol) and 14% BF_3_ (in methanol), following a previously described method [18]. One μL of FAME extract (in hexane) was injected into a gas chromatograph (6890N, Agilent Technologies, Inc., Santa Clara, CA, USA), equipped with an HP-Innowax column (30 m length × 0.32 mm I.D. × 0.25 μm film thickness, Agilent Technologies, Inc.) and autosampler (7683, Agilent Technologies, Inc.). Helium was used as a carrier gas at a constant flow rate of 1 mL/min. The injector and flame ionization detector temperatures were set to 260 and 280 °C, respectively. The oven temperature was programmed according to the following conditions: initial temperature of 150 °C, maintained for 1 min; increased from 150 to 200 °C at a rate of 15 °C/min; increased from 200 to 250 °C at a rate of 3 °C/min; and maintained at 250 °C for 5 min. The separated FAME peaks were identified by comparing their retention times with those of the commercially available standards. The results were expressed as the percentage (%) of individual fatty acid peak areas relative to the total peak area of fatty acids.

### 2.13. Statistical Analysis

Data were statistically analyzed using SPSS [19] Statistic version 21 A two-way analysis of variance (ANOVA) based on the general linear model was applied to evaluate the effects of slaughter weight and meat part, as well as their interaction, on the production of six major types of meat from the pig head. One-way ANOVA was used to estimate the effect of muscle part on proximate composition, energy content, collagen content, total myoglobin content, pH, WHC, purge loss, cooking loss, and fatty acid composition. When significant effects were detected, Duncan’s multiple range test was used to compare the mean values at *p* < 0.05.

## 3. Results

### 3.1. Pork Head Cut Yield

The absolute yields of the pork head and its six cuts from commercial crossbred pigs by slaughter weight group are presented in Table 2. The absolute yield of pork heads significantly increased with increasing slaughter weight. The 190–225 kg group had the highest value, approximately 2.4 times that of the 80–89 kg group. The absolute yields of pork head cuts typically increased with increasing slaughter weight. All pork head cuts had the highest absolute yields in the 190–225 kg group, whereas the 80–89 kg group had the lowest yields. The total meat yield was highest in the 190–225 kg group (2185.92 g), whereas the 80–89 and 90–99 kg groups had the lowest values (765.48 g and 883.26 g, respectively).

Within each slaughter weight groups, jowl meat had the highest absolute yield (*p* < 0.05). Occipital, cheek, and sublingual meats had significantly higher absolute yields than temporal and nasal meats (*p* < 0.05).

The relative yields of pork head and pork head cuts by slaughter weight group are presented in Table 3. There were no significant differences in relative yields among the slaughter weight groups for pork head or individual pork head cuts (*p* > 0.05). Within each slaughter weight group, jowl meat had the highest relative yield, and temporal and nasal meats had relatively lower yields (*p* < 0.05).

### 3.2. Proximate Composition, Energy Content, and Collagen Content

The proximate composition and the energy and collagen contents of the pork head cuts are presented in Table 4. The moisture content was lowest in occipital meat and highest in temporal and nasal meats (*p* < 0.05). Jowl meat had a lower moisture content than sublingual meat (*p* < 0.05).

Fat and energy contents were highest in occipital meat and lowest in cheek, temporal, nasal, and sublingual meats (*p* < 0.05). Protein content did not differ significantly among the pork head cuts (*p* > 0.05). Collagen content was significantly higher in occipital and jowl meats than in the other head cuts (*p* < 0.05).

### 3.3. pH, WHC, Purge Loss, and Cooking Loss

The pH, WHC, purge loss, and cooking loss values of the pork head cuts are presented in Table 5. Nasal meat had a significantly higher pH value than occipital, cheek, temporal, and jowl meats (*p* < 0.05). WHC was higher in jowl and occipital meats than in the other head cuts (*p* < 0.05). Purge loss differed significantly among cuts, with temporal meat showing the highest value and jowl meat showing the lowest value (*p* < 0.05). Cooking loss did not differ significantly among the pork head cuts (*p* > 0.05).

### 3.4. Total Myoglobin Content

The total myoglobin content differed significantly among the pork head cuts, ranging from 1.31 to 4.08 mg/g meat (Figure 3). Nasal, cheek, and sublingual meats had the highest myoglobin content followed by temporal, occipital, and jowl meats (*p* < 0.05).

### 3.5. CIE Color Values

The CIE color values of the pork head cuts are presented in Table 6. Jowl meat showed the highest L* value and the lowest a* value (*p* < 0.05). In contrast, sublingual and nasal meats showed lower L* values and higher a* values. Occipital meat also showed a relatively high L* value compared with nasal and sublingual meats (*p* < 0.05). Sublingual, occipital, and temporal meats showed higher b* values than jowl meat (*p* < 0.05).

Sublingual meat had a greater C* value than most other cuts, whereas jowl meat had a lower C* value (*p* < 0.05). The h° value was greatest in jowl meat and lowest in nasal and cheek meats (*p* < 0.05).

### 3.6. Fatty Acid Profiles

Across all cuts, C18:1n9 (oleic acid), C16:0 (palmitic acid), C18:0 (stearic acid) and C18:2n6 (linoleic acid) were the predominant fatty acids (Table 7). For saturated fatty acids (SFA), jowl meat had the greatest C16:0 proportion, whereas nasal meat had a lower total SFA proportion than most other cuts (*p* < 0.05). In contrast, the C18:0 proportion was lower in jowl meat than in temporal, occipital, and cheek meats (*p* < 0.05).

For monounsaturated fatty acids (MUFA), jowl meat showed a greater C16:1n7 (palmitoleic acid) proportion than the other cuts (*p* < 0.05). The C18:1n9 proportion was also higher in jowl meat than in cheek and sublingual meats, contributing to its higher total MUFA proportion. In contrast, the C20:1n9 (eicosenoic acid) proportion was relatively low in jowl meat.

For polyunsaturated fatty acids (PUFA), the proportion of C18:2n6 was significantly higher in nasal, occipital, and sublingual meats and lower in jowl meat (*p* < 0.05). Sublingual meat showed a significantly higher proportion of C18:3n3 (α-linolenic acid) than most other cuts (*p* < 0.05). The long-chain PUFA, including C20:4n6 and C22:4n6, were generally more abundant in cheek, temporal, nasal, and sublingual meats than in occipital and jowl meats (*p* < 0.05).

The UFA/SFA ratio was the highest in nasal meat (1.63; *p* < 0.05). Nasal and jowl meats showed greater MUFA/SFA ratios than cheek and sublingual meats (*p* < 0.05). The PUFA/SFA ratio was significantly higher in the nasal, sublingual, and occipital meats and lower in jowl meat (*p* < 0.05). The n6/n3 ratio was lower in jowl meat than in nasal, temporal, and cheek meats (*p* < 0.05).

## 4. Discussion

The present study demonstrated that yield differed according to slaughter weight, and that substantial differences in meat quality characteristics were evident among six pork head cuts from commercial crossbred pigs. The absolute yield increased with slaughter weight and varied significantly among cuts, whereas the relative yield remained stable across the slaughter weight groups. Additionally, fat and collagen content, WHC, meat color, and fatty acid profiles showed pronounced variations among the cuts.

### 4.1. Pork Head Cut Yield

Distinct differences were observed in the absolute yield of the pork head and its six individual cuts according to slaughter weight. Absolute yield increased significantly as slaughter weight increased, whereas relative yield remained primarily unchanged across slaughter weight groups. Notably, pigs in the 190–225 kg group exhibited a 2.4-fold greater head weight and 2.9-fold greater total head meat yield than those in the 80–89 kg group, indicating a substantial quantitative expansion of head tissues in heavier pigs. These findings are consistent with those of previous studies demonstrating that increasing slaughter or carcass weight results in higher absolute weights of primal and subprimal cuts [20,21,22,23]. Bertol et al. [20] observed that an increased slaughter weight was associated with a significant increase in the absolute weight of commercial pork cuts. The higher absolute yield of pork head cuts appeared to be primarily attributable to a concurrent increase in head weight. As slaughter weight increased, head weight also increased, indicating an increase in overall head size. Because individual head cuts are derived from the head as an anatomical unit, the expansion of the total head mass inevitably results in higher absolute cut weights. Accordingly, the observed increase in absolute yield indicates the quantitative enlargement of the head rather than the selective hypertrophy of specific tissues. This pattern is consistent with general carcass growth dynamics, in which increases in body weight are accompanied by proportional enlargement of anatomical structures [22]. From a biological perspective, this enlargement is likely to be mediated by growth-related changes in the skeletal muscle structure. Postnatal muscle growth in pigs is predominantly driven by muscle fiber hypertrophy rather than by hyperplasia [24]. Heavier pigs exhibit enhanced muscle fiber cross-sectional areas and, in certain cases, increased total fiber numbers, both of which contribute to enhanced muscle mass deposition [25,26,27]. Sustained hypertrophic growth is further supported by satellite cell activation and myonuclear accretion during postnatal development [24]. Therefore, the increased absolute yield observed in heavier slaughter weight groups may indicate macroscopic enlargement of the head, as well as cellular-level hypertrophic mechanisms underlying skeletal muscle growth.

In contrast, the relative yield did not differ significantly among the slaughter weight groups, suggesting that the proportional contribution of the head to total body weight remained stable across growth stages. As both head and overall slaughter weights increased simultaneously, the head-to-body weight ratio was maintained. Similar observations have been reported in studies evaluating the percentage yields of major pork cuts, where absolute cut weights increased with slaughter weight, whereas proportional yields exhibited limited variation [20,21,22]. Therefore, the stability of the relative yield indicates morphological scaling rather than disproportionate growth, demonstrating that the structural proportionality of the head within the overall body framework was preserved despite substantial increases in body mass.

To date, information regarding slaughter weight-dependent changes in the yield of individual pork head cuts remains limited. Although numerous studies have examined the effects of slaughter weight on commercial cuts, quantitative data on pork head cuts remain scarce. Consequently, a direct comparison with previous head-specific studies is constrained. However, the present findings provide novel baseline data for an underexplored anatomical region of the porcine head and support the applicability of established growth patterns in this region. In Korea, pork carcasses are officially classified into 25 standardized subprimary cuts [28]. When compared with the yields of these cuts, as presented in the national pork carcass yield standards of Korea [29], within the average slaughter weight range (111–120 kg), cheek (212.92 g) and jowl (330.62 g) meats exhibited absolute yields comparable to those of skirt meat (0.26–0.27 kg). Additionally, the combined absolute yield of all pork head cuts (1059.16 g) was within the reported range of tenderloin (1.26–1.31 kg), chuck tender (1.14–1.18 kg), eye of round (0.98–1.02 kg), and back ribs (1.03–1.10 kg). This quantitative comparison situates pork head cuts within the broader context of the pork carcass cutting system, and provides an objective reference for their relative scale among pork subprimal cuts.

### 4.2. Proximate Composition, Energy Content, and Collagen Content

The proximate composition of pork head meat has been reported only for cheek meat (*M. masseter*) in a limited number of studies [30,31]. In a study of Iberian pigs, Cava et al. [30] reported that the fat content of cheek meat (2.26 g/100 g meat) was lower than that of loin (*M. longissimus*, 4.79 g/100 g meat), whereas the protein content of cheek meat (21.31 g/100 g meat) was higher than that of loin (19.76 g/100 g meat). In contrast, compared with loin, Realini et al. [31] reported that cheek meat from Pietrain crossbred pigs contained a higher fat content (1.73 g/100 g meat) but a lower protein content (19.60 g/100 g meat) than that of loin (0.95 and 24.38 g/100 g meat, respectively). In the present study, the fat and protein contents of cheek meat were 4.12 and 19.96 g/100 g meat, respectively, which were slightly higher than those reported in previous studies [30,31]. These differences are likely attributable to variations in pig breeds and production systems, consistent with several previous studies [32,33,34,35]. NIAS [29] reported that the fat content of aldeungsimsal (Korean name for loin, *M. longissimus*) from the same commercial three-way crossbred pig (Landrace × Yorkshire × Duroc) breed as that used in the present study ranged from 4.48 to 5.30 g/100 g meat, whereas the protein content ranged from 23.22 to 23.52 g/100 g meat. Compared to those of aldeungsimsal, occipital and jowl meats showed higher fat and lower protein levels, whereas cheek, temporal, nasal, and sublingual meats showed lower or similar values.

Among pork head cuts, jowl meat corresponds to the same muscle as hangjeongsal (the Korean name for jowl meat) and is located in the forequarter region. According to NIAS [29], the fat content of hangjeongsal from three-way crossbred pigs (Landrace × Yorkshire × Duroc) ranged from 29.18 to 36.57 g/100 g meat, whereas the protein content ranged from 14.11 to 15.63 g/100 g meat. During the slaughter process, this muscle is divided when the head is removed, resulting in part of the muscle remaining attached to the head, whereas the remaining portion remains attached to the forequarter. However, the fat content of jowl meat observed in the present study was 3.2–3.6 times lower than that of hangjeongsals. This difference may be explained by the differences in sample preparation. In the present study, back fat was completely removed to facilitate an accurate comparison of the physicochemical characteristics of lean head meat, whereas in a previous study [29], back fat was not removed when preparing hangjeongsal samples according to commercial retail specifications. No previous studies have reported the proximate composition of occipital, temporal, nasal, and sublingual meats. However, the differences in fat content observed among pork head cuts in the present study may be attributed to the variation in intramuscular fat content based on the anatomical muscle origin [36].

The inverse relationship observed between moisture and fat content in pork head meat in the present study is consistent with patterns reported for other pork cuts [37,38,39]. In general, as lipid deposition increases in muscle tissue, the relative proportion of water decreases because fat partially replaces the aqueous fraction of the muscle [40].

In the present study, differences in the energy content among pork head meat followed the same pattern as differences in the fat content, with occipital and jowl meat showing the highest energy values. This is likely because the protein content of pork head meat was similar, whereas the fat content differed notably depending on the anatomical location, with occipital and jowl meats showing the highest lipid levels. The elevated energy values associated with increased fat content observed in the present study are consistent with previous studies, indicating that the energy content in meat products is predominantly determined by fat content [41,42,43].

Studies on the collagen content of pork head meat are notably limited. Realini et al. [31] reported that in Pietrain crossbred pigs, cheek meat (*M. masseter*, 2.09 g/100 g meat) contained a higher collagen content than that of loin (*M. longissimus*, 0.54) and eye of round (*M. semitendinosus*, 0.85). However, the collagen content of occipital, jowl, temporal, nasal, and sublingual meats has not been previously reported. In a study on three-way crossbred pigs (Landrace × Yorkshire × Duroc), NIAS [29] reported that collagen contents of aldeungsimsal and hongdukkaesal (Korean name for eye of round) ranged from 0.99 to 1.37 g/100 g meat and from 1.40 to 1.65 g/100 g meat, respectively. In the present study, the collagen content of the cheek meat did not differ significantly from the values reported for aldeungsimsal and hongdukkaesal, in contrast to the findings reported by Realini et al. [31]. This difference may be attributed to the influence of collagen content in pork by the pig breed [44,45].

In the present study, occipital and jowl meats showed substantially higher collagen levels than those of cheek, temporal, nasal, and sublingual meats. Previous studies have reported that adipose tissue develops within connective tissue compartments of skeletal muscle [46,47]. Intramuscular fat is primarily deposited within the perimysial connective tissue network; therefore, muscles with increased lipid accumulation may exhibit more developed connective tissue structures [46]. As collagen is the major structural component of the intramuscular connective tissue, muscles with increased connective tissue development tend to have higher collagen content [48,49]. Therefore, the notably higher collagen content observed in the occipital and jowl meat in the present study may be associated with increased connective tissue development accompanied by higher fat deposition in these anatomical regions.

### 4.3. pH, WHC, Purge Loss, and Cooking Loss

The ultimate pH of pork typically ranges from 5.6 to 5.9 [50]. In this study, the pH of pork head meat ranged from 5.86 to 6.23, with nasal meat showing the highest pH value. Following slaughter, the muscle pH gradually declines as lactic acid accumulates through anaerobic glycolysis [51]. Therefore, the final pH of meat is determined by the extent of postmortem glycolysis, which may vary among muscles and meat cuts.

In certain pork head cuts, the muscle fiber characteristics may have influenced the pH values. In particular, cheek meat (*M. masseter*) consists predominantly of type I and type IIA muscle fibers, which are associated with oxidative metabolism [31,52]. England et al. [52] reported that the oxidative *M. master* exhibited a lower glycolytic capacity and, consequently, a higher ultimate pH than that of the glycolytic *M. longissimus lumborum*. Similar to cheek meat, nasal and sublingual meats also showed relatively high a* values and myoglobin content, a pattern consistent with the characteristics of oxidative muscles [36]. Therefore, the high pH values of nasal and sublingual meats may be partly attributable to oxidative muscle characteristics.

In contrast, jowl meat had a numerically lower pH than that of the other head cuts, and its pH was similar to the range of 5.84–5.88 reported for hangjeongsal by NIAS [29]. In this study, jowl meat had a higher L* value, lower a* value, and lower myoglobin content than those of the other head cuts. These characteristics are typical of glycolytic white muscle [36].

White muscles are predominantly composed of glycolytic muscle fibers and exhibit a lower final pH owing to rapid postmortem lactic acid accumulation [31,52]. However, the pH of jowl meat did not differ significantly from that of other pork head cuts, except for nasal meat. This finding suggests that the differences in the ultimate pH of pork head meat cannot be explained solely by muscle fiber characteristics.

Previous studies directly comparing the pH values of individual pork head cuts are limited, and information on the pH of nasal, occipital, temporal, and sublingual meat is particularly limited. The differences observed between pork head cuts in the present study likely indicate the combined effects of multiple factors, including muscle metabolic characteristics, glycolytic potential, buffering capacity, pre-slaughter conditions, sample preparation, and cut-specific tissue composition [51]. Further studies examining muscle fiber composition and postmortem metabolic changes are needed to elucidate the mechanisms underlying cut-specific differences in the ultimate pH. However, the present findings provide valuable baseline information on the pH characteristics of individual pork head cuts.

The contrasting WHC and purge loss patterns observed in temporal and occipital meats may be related to their pH values. Generally, as meat pH decreases toward the isoelectric point of muscle proteins, the net charge of proteins and electrostatic repulsion between myofibrils decrease, reducing the space available for water retention [53]. Additionally, changes in pH may also reduce the water-binding capacity of proteins, thereby decreasing WHC. Accordingly, the relatively low pH of temporal meat may have contributed to its reduced WHC and increased purge loss, whereas the higher pH of occipital meat may partly account for its increased WHC and reduced purge loss.

However, the pH and water-holding properties did not follow the same pattern for all pork head cuts. In particular, jowl meat had a relatively low pH; however, it exhibited a significantly increased WHC and decreased purge loss. This suggests that the water-holding properties of pork head meat cannot be explained by pH alone. Although meat WHC is strongly affected by pH, water loss can also be influenced by temperature, muscle structure, protein denaturation, water distribution, and meat composition [53,54]. The low purge loss of jowl meat may be associated with its low moisture content. Previous studies have reported that pork with a high fat and low moisture content can have reduced water loss characteristics, particularly a lower cooking loss [55,56]. Therefore, meat containing less water may also have less water available for release, resulting in a lower purge loss.

The cooking loss did not differ significantly among the six pork head cuts. Cooking loss is influenced by the pH and WHC of fresh meat, as well as by cooking conditions and heat-induced changes, including protein denaturation, muscle fiber shrinkage, and water redistribution [57,58]. Therefore, although differences in WHC and purge loss were observed among the head meat samples, changes induced by heating may have attenuated the differences in fresh meat.

### 4.4. Total Myoglobin Content

Myoglobin is the primary pigment responsible for the red color of meat [59]. Significant differences in myoglobin content were observed among different pork head meats. Nasal, sublingual, and cheek meats had significantly higher myoglobin content, whereas jowl meat had the lowest content. These differences were consistent with the trends observed in the a* values of each cut, suggesting that differences in total myoglobin content partially contributed to meat redness.

The variation in myoglobin content may be associated with the metabolic characteristics and muscle fiber composition of the muscles in each head meat. Type I and type IIA muscle fibers have predominantly oxidative metabolic properties and typically contain high concentrations of myoglobin, resulting in a red color [36]. In particular, the cheek meat (*M. masseter*) is an oxidative muscle composed mainly of type I and type IIA fibers [31,52]. These characteristics may have contributed to the high myoglobin content in cheek meat. The relatively high a* values and myoglobin content of the nasal and sublingual meats also indicate that these cuts may exhibit characteristics that are more typical of red and oxidative muscles.

In contrast, the low myoglobin content of jowl meat may indicate muscle characteristics that are more typical of white glycolytic muscles. These muscles rely predominantly on glycolytic metabolism and have relatively low oxygen requirements; therefore, they typically contain less myoglobin than oxidative muscles [36]. However, this study did not analyze the muscle fiber composition, and previous studies on the fiber characteristics of individual pork head meats remain limited. Therefore, further studies are required to elucidate the relationship between muscle fiber composition, myoglobin content, and meat color in pork head cuts.

### 4.5. CIE Color Values

Jowl meat had the highest L* and lowest a* values among the pork head cuts. This result was visually consistent with that shown in Figure 2, in which the jowl meat appeared lighter and paler than those of the other cuts. The L* value of hangjeongsal (the same cut as jowl meat) reported by the NIAS ranged from 63.95 to 67.07, and the a* value ranged from 8.51 to 8.97 [29]. These values indicate relatively high lightness and low redness, which is consistent with the color characteristics of jowl meat observed in the present study.

In contrast, nasal and sublingual meats showed significantly lower L* values and higher a* values. These results may be associated with the pH values. The two cuts had relatively high pH values of 6.23 and 6.04. Previous studies have reported a negative relationship between meat pH and L* values [50,60]. At a higher pH, swelling of the myofibrillar structure lengthens the path of light passage, absorbs more light, and reduces light scattering and surface reflectance, resulting in a darker appearance [60].

Additionally, a higher pH may improve color stability by delaying myoglobin oxidation and metmyoglobin formation, whereas acidic conditions may accelerate myoglobin oxidation, leading to meat discoloration [61]. Therefore, the relatively high pH values of the nasal and sublingual meat may have contributed to their darker and redder appearances. In addition, as discussed above, nasal, sublingual, and cheek meats had relatively high total myoglobin contents, consistent with their high a* values.

The occipital meat had a significantly higher L* value than that of the nasal and sublingual meats, and also had a high b* value. These characteristics may be due to the high fat content of the occipital meat. Utrilla et al. [62] reported that pork loins with a higher fat content had higher L* and b* values. The light color and slightly yellow appearance of fat may have contributed to the L* and b* values of occipital meat.

Chroma (C*) values represent color saturation and are determined from the a* and b* values [63]. In this study, the sublingual meat had the highest C* value, which was consistent with its relatively high a* and b* values. In contrast, the jowl meat had the lowest C* value, indicating a less saturated color. The hue angle (h°) represents the development of color from red to yellow, with a high angle indicating reduced redness [63]. Jowl meat had the highest h° value, which was consistent with its low a* value. The nasal meat had the lowest h° value because of its relatively low b*/a* ratio.

Overall, the pork head meats exhibited distinct color characteristics depending on the cut. These differences likely indicate the combined effects of pH, water-holding properties, fat and myoglobin contents, myoglobin stability, and muscle fiber characteristics. This suggests that the individual pork head meat should be evaluated as a distinct meat cut with distinct characteristics.

### 4.6. Fatty Acid Profiles

The major fatty acids in pork head meats were oleic acid (C18:1n9), palmitic acid (C16:0), linoleic acid (C18:2n6), and stearic acid (C18:0), which are similar to the major fatty acid composition of pork loin muscle reported previously [32,64,65]. The proportion of certain fatty acids varied among the pork head meat samples.

Nasal and sublingual meats had significantly higher levels of linoleic acid (C18:2n6) and total PUFA, resulting in a higher PUFA/SFA ratio. As indicated by their color and myoglobin content, these cuts may contain muscles with relatively oxidative metabolic characteristics. Oxidative muscles typically have a higher proportion of membrane phospholipids and mitochondrial density than that of glycolytic muscles [31,65,66]. Membrane phospholipids contain relatively high proportions of PUFA [65,66]. Therefore, the fatty acid profiles of nasal and sublingual meats may indicate the metabolic characteristics of the muscles present in these cuts. Although cheek meat did not have the highest proportion of PUFAs, its PUFA content was higher than that of jowl meat. Cheek meat also contained high levels of long-chain PUFAs, including C20:4n6 and C22:4n6. The masseter muscle is composed of oxidized muscle fiber [31,52]. Therefore, the fatty acid profile of the cheek meat may be influenced by muscle characteristics.

In contrast, jowl meat had significantly higher oleic acid (C18:1n9) and MUFA contents, whereas the PUFA content was significantly lower. This may be due to the high fat content and muscle characteristics of jowl meat, which are similar to those of white meat. Jowl meat had a low myoglobin content and a light, pale color, imparting characteristics similar to white meat. White meat is mainly composed of glycolytic muscle fibers and has a lower phospholipid content than that of oxidative muscle [31]. In addition, meat containing an increased amount of fat, particularly neutral lipids, generally contains higher oleic acid and lower PUFA [65]. These characteristics may have contributed to the high proportions of MUFA and PUFA observed in the jowl meat. Occipital meat had a high fat content and low myoglobin content, similar to jowl meat, but with a relatively high PUFA proportion. This contrasting result suggests that the fatty acid composition of pork head meat cannot be explained solely by the total fat content or muscle fiber characteristics. Various other factors, such as the proportions of neutral and phospholipids, anatomical location, and local lipid metabolism, may also contribute to differences in fatty acid composition [65].

The n6/n3 fatty acid ratio is an important attribute from a health and nutritional perspective [32,64]. The ideal n6/n3 ratio is less than 4. However, all pork head meat samples exhibited high n6/n3 ratios. Pork commonly has a high linoleic acid content because pigs are frequently fed grain-based diets rich in n-6 fatty acids [32,64]. Therefore, the high n-6: n-3 ratios observed in the present study may primarily indicate the fatty acid composition of the diet provided to pigs.

Overall, the present findings demonstrated that individual pork head cuts have distinct fatty acid profiles. These differences may indicate the combined effects of fat deposition, lipid class composition, muscle metabolic characteristics, and other cut-specific factors. These results provide baseline information for evaluating the nutritional characteristics and potential utilization of individual pork head cuts.

## 5. Conclusions

This study investigated changes in the yields of pork heads and six individual pork head cuts across slaughter weight groups and compared the physicochemical properties of the six pork head cuts. As the slaughter weight increased, the absolute yields of the pork head and six pork head cuts increased; however, the relative yields did not differ significantly. This indicated that the increase in pork head meat production with increasing slaughter weight was mainly attributable to quantitative enlargement of the head rather than to selective hypertrophy of specific tissues.

Physicochemical properties differed notably among the six cuts. Nasal and sublingual meats had significantly higher pH, redness, and myoglobin content, whereas jowl meat had high fat content, higher L* value, and low myoglobin content. Occipital, cheek, and temporal meats also showed cut-specific quality properties that distinguished them from other cuts.

These results may provide practical baseline information for the utilization of pork head cuts as edible meat resources. The yield data can help processors estimate the expected amounts of raw materials obtained from pigs within different slaughter weight ranges. In addition, the distinct quality characteristics of each cut suggest that pork head cuts may be classified and selected according to their specific properties rather than being used only as a mixed raw material. The fatty acid composition data also provide basic information for describing the nutritional characteristics of individual cuts. However, because this study mainly evaluated the basic quality properties of each cut, further studies on storage stability and processing properties are required to improve their utilization.

## Figures and Tables

**Figure 1:**
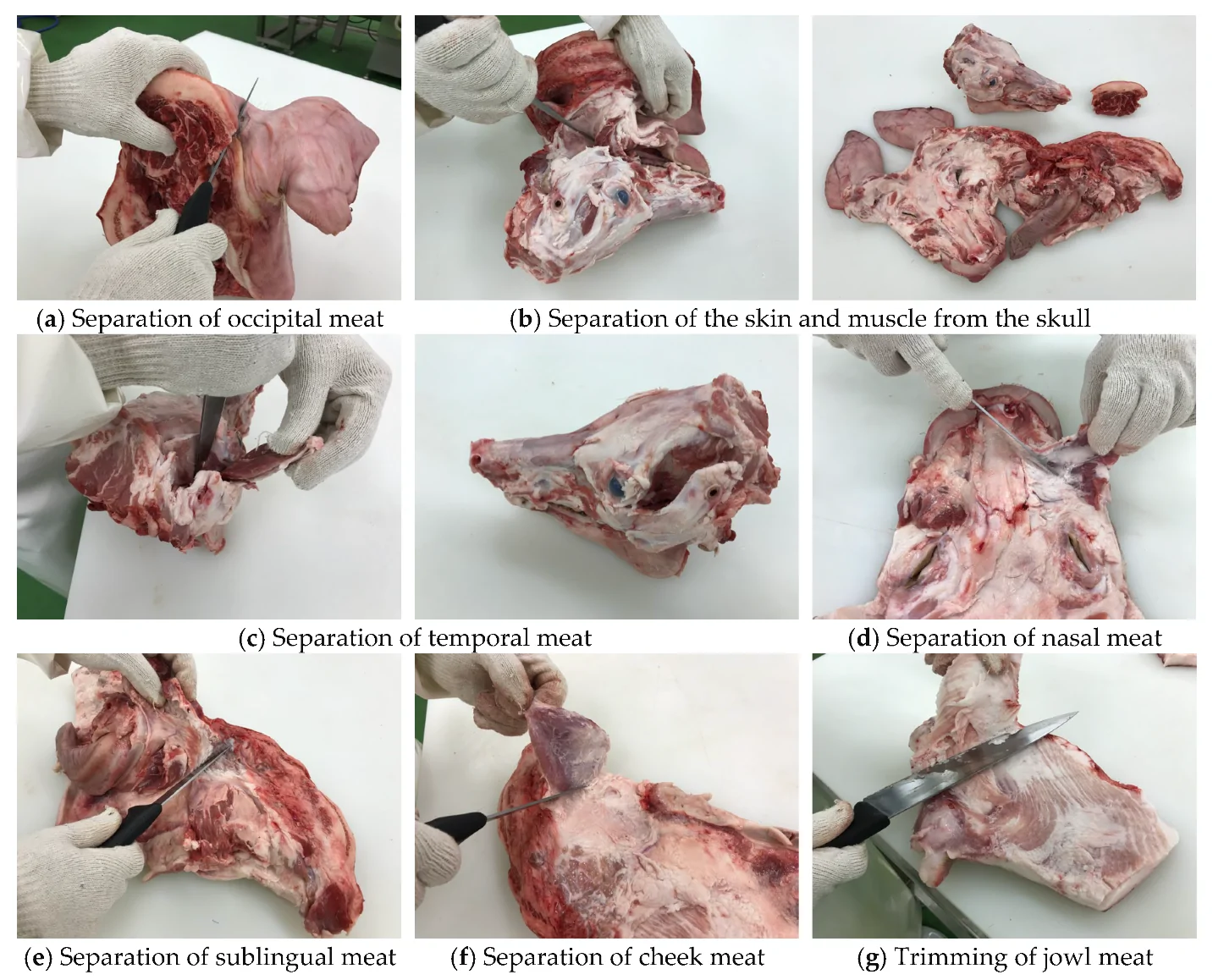
Fabrication procedures for six pork head cuts.

**Figure 2:**
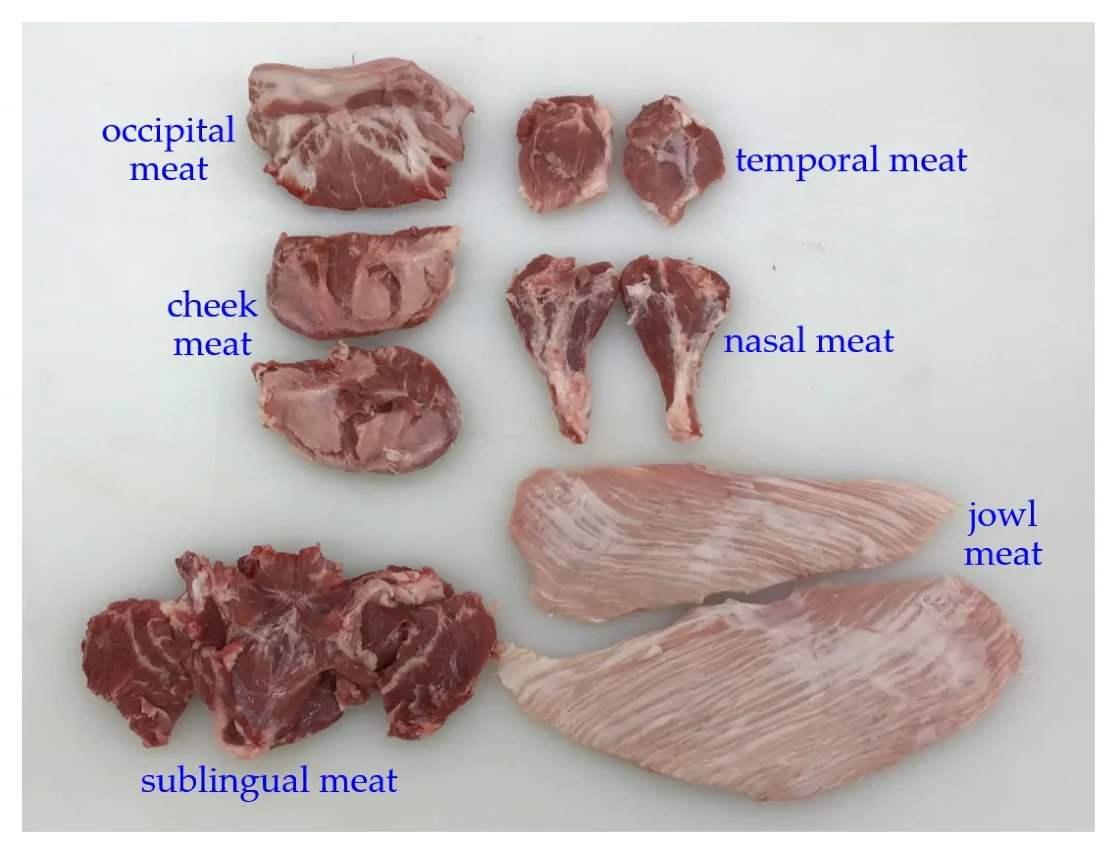
Final appearance of six pork head cuts.

**Figure 3:**
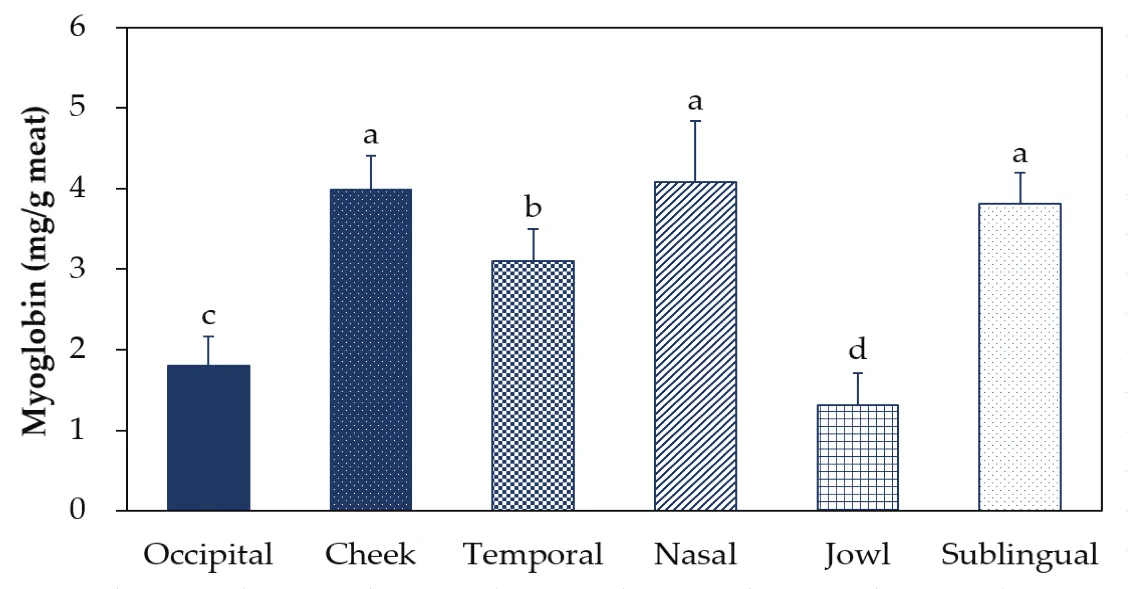
The total myoglobin content of pork head cuts from commercial crossbred pigs (Landrace × Yorkshire × Duroc). These data are presented as means ± standard deviation. ^a–d^ Different letters indicate significant differences among pork head cuts (*p* < 0.05).

**Table 1:** Characteristics of experimental animals by slaughter weight group.

Slaughter Weight Group (kg)	Animal (*n*)	Average Weight ^1^ (kg)	Sex Distribution (*n*)	
			Castrated Male	Female
80–89	10	85.30 ± 1.95	4	6
90–99	25	94.36 ± 2.78	8	17
100–109	35	105.30 ± 3.05	28	7
110–119	62	113.63 ± 2.61	52	10
120–135	8	124.31 ± 5.73	4	4
190–225	10	213.20 ± 9.77	-	10 ^2^

^1^ Values are presented as means ± standard deviation. ^2^ Culled breeding sows.

**Table 2:** The absolute yields of six pork head cuts from commercial crossbred pigs (Landrace × Yorkshire × Duroc) by slaughter weight group.

Item	Slaughter Weight Group (kg)					
	80–89	90–99	100–109	110–119	120–135	190–225
Head (kg)	5.16 ± 0.42 ^e^	5.74 ± 0.58 ^d^	6.50 ± 0.63 ^c^	6.91 ± 0.60 ^bc^	7.15 ± 0.52 ^b^	12.58 ± 0.67 ^a^
Occipital (g)	120.72 ± 31.79 ^c Y^	132.02 ± 70.27 ^c Y^	163.42 ± 53.17 ^bc Y^	178.73 ± 54.84 ^b Y^	151.47 ± 59.11 ^bc Y^	272.93 ± 63.06 ^a Y^
Cheek (g)	165.56 ± 31.40 ^d X^	190.79 ± 29.53 ^cd X^	207.57 ± 29.17 ^bc X^	212.92 ± 28.83 ^bc X^	232.72 ± 45.66 ^b X^	445.77 ± 100.66 ^a X^
Temporal (g)	62.69 ± 12.42 ^c Z^	67.91 ± 16.69 ^bc Z^	68.29 ± 13.76 ^bc Z^	73.65 ± 18.36 ^bc Z^	80.87 ± 21.78 ^b Z^	189.23 ± 33.26 ^a Z^
Nasal (g)	76.04 ± 16.00 ^c Z^	81.71 ± 12.40 ^c Z^	84.90 ± 13.60 ^bc Z^	88.26 ± 14.57 ^bc Z^	97.13 ± 18.50 ^bY Z^	183.03 ± 33.57 ^a Z^
Jowl (g)	211.51 ± 77.70 ^d W^	245.21 ± 92.62 ^cd W^	322.80 ± 101.74 ^bc W^	330.62 ± 119.03 ^b W^	359.04 ± 104.58 ^b W^	669.39 ± 153.33 ^a W^
Sublingual (g)	128.96 ± 20.13 ^c Y^	165.61 ± 39.34 ^b X^	180.51 ± 51.42 ^b Y^	174.98 ± 48.28 ^b Y^	199.96 ± 32.50 ^b XY^	425.58 ± 87.14 ^a X^
Total ^1^ (g)	765.48 ± 114.31 ^c^	883.26 ± 158.15 ^c^	1027.50 ± 185.86 ^b^	1059.16 ± 186.20 ^b^	1121.18 ± 165.39 ^b^	2185.92 ± 317.34 ^a^

These data are presented as means ± standard deviation. ^a–e^ Different letters within the same row indicate significant differences among slaughter weight groups (*p* < 0.05). ^W–Z^ Different letters within the same column indicate significant differences among pork head cuts (*p* < 0.05). ^1^ Total values represent the sum of the six head cut yields.

**Table 3:** The relative yields of six pork head cuts from commercial crossbred pigs (Landrace × Yorkshire × Duroc) by slaughter weight group.

Item	Slaughter Weight Group (kg)					
	80–89	90–99	100–109	110–119	120–135	190–225
Head (%)	6.05 ± 0.43	6.08 ± 0.56	6.17 ± 0.56	6.09 ± 0.52	5.75 ± 0.40	5.91 ± 0.31
Occipital (%)	0.14 ± 0.04 ^Y^	0.14 ± 0.08 ^Y^	0.16 ± 0.05 ^Y^	0.16 ± 0.05 ^Y^	0.12 ± 0.05 ^Y^	0.13 ± 0.03 ^Y^
Cheek (%)	0.19 ± 0.04 ^X^	0.20 ± 0.03 ^X^	0.20 ± 0.03 ^X^	0.19 ± 0.03 ^X^	0.19 ± 0.04 ^X^	0.21 ± 0.05 ^X^
Temporal (%)	0.07 ± 0.01 ^Z^	0.07 ± 0.02 ^Z^	0.06 ± 0.01 ^Z^	0.06 ± 0.02 ^Z^	0.06 ± 0.02 ^Z^	0.09 ± 0.02 ^Z^
Nasal (%)	0.09 ± 0.02 ^Z^	0.09 ± 0.01 ^Z^	0.08 ± 0.01 ^Z^	0.08 ± 0.01 ^Z^	0.08 ± 0.01 ^Z^	0.09 ± 0.02 ^Z^
Jowl (%)	0.25 ± 0.09 ^W^	0.26 ± 0.10 ^W^	0.31 ± 0.10 ^W^	0.29 ± 0.10 ^W^	0.29 ± 0.08 ^W^	0.31 ± 0.07 ^W^
Sublingual (%)	0.15 ± 0.02 ^Y^	0.18 ± 0.04 ^X^	0.17 ± 0.05 ^Y^	0.15 ± 0.04 ^Y^	0.16 ± 0.02 ^XY^	0.20 ± 0.04 ^X^
Total ^1^ (%)	0.90 ± 0.13	0.94 ± 0.17	0.98 ± 0.18	0.93 ± 0.16	0.90 ± 0.13	1.03 ± 0.15

These data are presented as means ± standard deviation. ^W–Z^ Different letters within the same column indicate significant differences among pork head cuts (*p* < 0.05). ^1^ Total values represent the sum of the six head cut yields.

**Table 4:** The proximate composition and energy and collagen contents of six pork head cuts from commercial crossbred pigs (Landrace × Yorkshire × Duroc).

Item	Pork Head Cut					
	Occipital	Cheek	Temporal	Nasal	Jowl	Sublingual
Moisture (g/100 g meat)	67.09 ± 2.25 ^d^	76.52 ± 1.50 ^ab^	77.25 ± 1.33 ^a^	77.38 ± 1.49 ^a^	69.97 ± 2.15 ^c^	75.57 ± 1.10 ^b^
Fat (g/100 g meat)	12.27 ± 3.76 ^a^	4.12 ± 1.07 ^c^	3.41 ± 0.99 ^c^	3.02 ± 1.21 ^c^	9.62 ± 3.01 ^b^	4.75 ± 0.93 ^c^
Protein (g/100 g meat)	20.89 ± 1.37	19.96 ± 0.68	20.15 ± 0.89	20.64 ± 0.80	20.54 ± 1.16	19.98 ± 0.89
Energy (kcal/100 g meat)	199.88 ± 28.34 ^a^	122.39 ± 9.70 ^c^	116.80 ± 9.98 ^c^	115.37 ± 10.06 ^c^	174.48 ± 24.12 ^b^	128.16 ± 9.89 ^c^
Collagen (g/100 g meat)	3.02 ± 0.27 ^a^	1.50 ± 0.04 ^b^	1.19 ± 0.04 ^b^	1.59 ± 0.08 ^b^	2.98 ± 0.09 ^a^	1.24 ± 0.10 ^b^

These data are presented as means ± standard deviation. ^a–d^ Different letters indicate significant differences among pork head cuts (*p* < 0.05).

**Table 5:** The pH, water-holding capacity, purge loss, and cooking loss of six pork head cuts from commercial crossbred pigs (Landrace × Yorkshire × Duroc).

Item	Pork Head Cut					
	Occipital	Cheek	Temporal	Nasal	Jowl	Sublingual
pH	6.01 ± 0.33 ^b^	5.97 ± 0.10 ^b^	5.86 ± 0.08 ^b^	6.23 ± 0.15 ^a^	5.88 ± 0.28 ^b^	6.04 ± 0.15 ^ab^
WHC ^1^ (%)	78.47 ± 5.10 ^a^	66.00 ± 3.12 ^b^	65.89 ± 3.33 ^b^	70.99 ± 4.64 ^b^	78.99 ± 7.66 ^a^	69.32 ± 3.42 ^b^
Purge loss (%)	1.76 ± 0.60 ^c^	2.19 ± 0.20 ^c^	4.45 ± 1.39 ^a^	3.49 ± 1.39 ^b^	1.43 ± 0.48 ^c^	3.20 ± 0.76 ^b^
Cooking loss (%)	20.61 ± 1.93	24.68 ± 2.80	24.08 ± 3.78	24.82 ± 4.73	22.65 ± 4.37	21.45 ± 2.90

These data are presented as means ± standard deviation. ^a–c^ Different letters indicate significant differences among pork head cuts (*p* < 0.05). ^1^ Water-holding capacity.

**Table 6:** The CIE L*, a*, b*, C*, and h° values of six pork head cuts from commercial crossbred pigs (Landrace × Yorkshire × Duroc).

Item	Pork Head Cut					
	Occipital	Cheek	Temporal	Nasal	Jowl	Sublingual
L* (lightness)	47.44 ± 2.59 ^b^	44.24 ± 2.33 ^bc^	45.77 ± 1.85 ^bc^	41.76 ± 3.26 ^d^	57.75 ± 3.21 ^a^	42.79 ± 4.51 ^cd^
a* (redness)	14.07 ± 1.91 ^c^	16.74 ± 1.00 ^ab^	15.69 ± 1.09 ^b^	17.57 ± 1.38 ^a^	7.97 ± 1.09 ^d^	17.78 ± 2.30 ^a^
b* (yellowness)	7.05 ± 1.34 ^ab^	5.94 ± 0.86 ^bc^	6.90 ± 1.14 ^ab^	5.95 ± 1.17 ^bc^	5.38 ± 0.84 ^c^	7.23 ± 1.40 ^a^
C* (chroma)	15.81 ± 2.09 ^c^	17.79 ± 1.06 ^ab^	17.18 ± 1.29 ^bc^	18.59 ± 1.46 ^ab^	9.68 ± 0.94 ^d^	19.27 ± 2.44 ^a^
h° (hue-angle)	26.73 ± 3.86 ^b^	19.49 ± 2.54 ^d^	23.68 ± 2.96 ^bc^	18.62 ± 3.36 ^d^	34.10 ± 6.09 ^a^	22.20 ± 3.42 ^cd^

These data are presented as means ± standard deviation. ^a–d^ Different letters indicate significant differences among pork head cuts (*p* < 0.05).

**Table 7:** Fatty acid profiles of six pork head cuts from commercial crossbred pigs (Landrace × Yorkshire × Duroc).

Item	Pork Head Cut					
	Occipital	Cheek	Temporal	Nasal	Jowl	Sublingual
C14:0 (%)	1.52 ± 0.14	1.57 ± 0.19	1.55 ± 0.14	1.37 ± 0.29	1.63 ± 0.14	1.47 ± 0.30
C16:0 (%)	25.31 ± 0.49 ^b^	26.67 ± 0.87 ^ab^	26.64 ± 1.03 ^ab^	25.29 ± 1.61 ^b^	27.87 ± 2.27 ^a^	26.01 ± 0.56 ^b^
C16:1n7 (%)	1.72 ± 0.44 ^b^	1.79 ± 0.27 ^b^	2.00 ± 0.50 ^b^	2.05 ± 0.39 ^b^	2.61 ± 0.86 ^a^	1.84 ± 0.44 ^b^
C18:0 (%)	13.02 ± 1.12 ^ab^	13.50 ± 1.24 ^a^	12.97 ± 0.60 ^ab^	11.46 ± 1.06 ^bc^	11.07 ± 3.64 ^c^	12.85 ± 0.60 ^abc^
C18:1n9 (%)	41.66 ± 1.71 ^ab^	41.44 ± 2.89 ^b^	42.12 ± 3.35 ^ab^	42.63 ± 2.99 ^ab^	44.69 ± 3.25^a^	39.61 ± 2.69 ^b^
C18:1n7 (%)	0.18 ± 0.02	0.17 ± 0.04	0.16 ± 0.03	0.17 ± 0.04	0.18 ± 0.05	0.18 ± 0.03
C18:2n6 (%)	14.74 ± 1.30 ^a^	12.58 ± 1.79 ^b^	12.44 ± 2.30 ^b^	14.80 ± 0.81 ^a^	10.53 ± 1.83 ^c^	15.50 ± 1.51 ^a^
C18:3n3 (%)	0.55 ± 0.07 ^ab^	0.37 ± 0.09 ^c^	0.42 ± 0.10 ^c^	0.51 ± 0.06 ^b^	0.42 ± 0.08 ^c^	0.61 ± 0.10 ^a^
C20:1n9 (%)	0.80 ± 0.07 ^ab^	0.96 ± 0.11 ^a^	0.90 ± 0.11 ^abc^	0.82 ± 0.08 ^bcd^	0.77 ± 0.11 ^d^	0.93 ± 0.17 ^ab^
C20:4n6 (%)	0.37 ± 0.07 ^b^	0.75 ± 0.32 ^a^	0.63 ± 0.25 ^a^	0.71 ± 0.28 ^a^	0.28 ± 0.08 ^b^	0.67 ± 0.23 ^a^
C22:4n6 (%)	0.13 ± 0.02 ^b^	0.21 ± 0.05 ^a^	0.17 ± 0.04 ^a^	0.18 ± 0.03 ^a^	0.08 ± 0.02 ^c^	0.18 ± 0.07 ^a^
SFA ^1^ (%)	39.85 ± 1.44 ^ab^	41.73 ± 1.63 ^a^	41.16 ± 1.49 ^a^	38.13 ± 2.22 ^b^	40.42 ± 2.69 ^a^	40.48 ± 1.05 ^a^
UFA ^2^ (%)	60.15 ± 1.44 ^ab^	58.27 ± 1.63 ^b^	58.84 ± 1.49 ^b^	61.87 ± 2.22 ^a^	59.58 ± 2.69 ^b^	59.52 ± 1.05 ^b^
MUFA ^3^ (%)	44.36 ± 1.79 ^bc^	44.36 ± 2.86 ^bc^	45.18 ± 2.90 ^bc^	45.67 ± 2.93 ^ab^	48.25 ± 2.95 ^a^	42.56 ± 2.36 ^c^
PUFA ^4^ (%)	15.79 ± 1.36 ^a^	13.91 ± 1.96 ^b^	13.67 ± 2.56 ^b^	16.20 ± 0.94 ^a^	11.32 ± 1.94 ^c^	16.96 ± 1.71 ^a^
UFA/SFA	1.51 ± 0.09 ^b^	1.40 ± 0.09 ^b^	1.43 ± 0.09 ^b^	1.63 ± 0.15 ^a^	1.48 ± 0.17 ^b^	1.47 ± 0.06 ^b^
MUFA/SFA	1.11 ± 0.08 ^ab^	1.07 ± 0.10 ^b^	1.10 ± 0.10 ^ab^	1.21 ± 0.14 ^a^	1.20 ± 0.15 ^a^	1.05 ± 0.08 ^b^
PUFA/SFA	0.40 ± 0.04 ^a^	0.33 ± 0.05 ^b^	0.33 ± 0.06 ^b^	0.43 ± 0.02 ^a^	0.28 ± 0.05 ^c^	0.42 ± 0.04 ^a^
n6/n3	27.95 ± 1.83 ^cd^	37.10 ± 5.50 ^a^	32.19 ± 3.44 ^b^	30.81 ± 3.97 ^bc^	26.37 ± 3.98 ^d^	27.17 ± 3.54 ^cd^

These data are presented as means ± standard deviation. ^a–d^ Different letters indicate significant differences among pork head cuts (*p* < 0.05). ^1^ Saturated fatty acids. ^2^ Unsaturated fatty acids. ^3^ Monounsaturated fatty acids. ^4^ Polyunsaturated fatty acids.

## Data Availability

All data generated or analyzed during this study are included in this published article.

## References

[1] ORCD/FAO (2024). OECD-FAO Agricultural Outlook 2024–2033.

[2] Korea Rural Economic Institute (2026). Agricultural Outlook 2026.

[3] Al-Zohairi S., Knudsen M.T., Mogensen L. (2023). Utilizing animal by-products in European slaughterhouses to reduce the environmental footprint of pork products. Sustain. Prod. Consump..

[4] Toldrá F., Aristoy M.C., Mora L., Reig M. (2012). Innovations in value-addition of edible meat by-products. Meat Sci..

[5] Seong P.N., Park K.M., Cho S.H., Kang S.M., Kang G.H., Park B.Y., Moon S.S., Van Ba H. (2014). Characterization of edible pork by-products by means of yield and nutritional composition. Korean J. Food Sci. Anim. Resour..

[6] Choi Y.S., Hwang K.E., Kim H.W., Song D.H., Jeon K.H., Park J.D., Sung J.M., Kim Y.B., Kim C.J. (2016). Replacement of pork meat with pork head meat for frankfurters. Korean J. Food Sci. Anim. Resour..

[7] Choi Y.S., Jeon K.H., Ku S.K., Sung J.M., Choi H.W., Seo D.H., Kim C.J., Kim Y.B. (2016). Quality characteristics of replacing pork hind leg with pork head meat for hamburger patties. Korean J. Food Cook. Sci..

[8] MFDS (2021). Enforcement Rule of the Livestock Products Sanity Control Act.

[9] Anderson S. (2007). Determination of fat, moisture, and protein in meat and meat products by using the FOSS FoodScan near-infrared spectrophotometer with FOSS artificial neural network calibration model and associated da-tabase: Collaborative study. J. AOAC Int..

[10] FAO (2003). Food Energy–Methods of Analysis and Conversion Factors.

[11] Suman S.P., Mancini R.A., Joseph P., Ramanathan R., Konda M.K.R., Dady G., Naveena B.M., López-López I. (2010). Color-stabilizing effect of lactate on ground beef is packaging-dependent. Meat Sci..

[12] Fischer C., Hofmann K., Hamm R. (1976). Erfahrungen mit der Kapillarvolumeter-Methode nach Hofmann zur Bestimmung des Wassebindungsvermögens von Fleisch. Fleischwirtschaft.

[13] Holmer S.F., McKeith F.K., Killefer J. (2008). The influence of early post-mortem enhancement and accelerated chilling on pork quality. Meat Sci..

[14] Honikel K.O. (1998). Reference methods for assessment of physical characteristics of meat. Meat Sci..

[15] Sammel L.M., Hunt M.C., Kropf D.H., Hachmeister K.A., Johnson D.E. (2002). Comparison of assays for metmyoglobin reducing ability in beef inside and outside semimembranosus muscle. J. Food Sci..

[16] CIE (1986). Colorimetry: Official Recommendations of the International Commission on Illumination.

[17] Folch J.M., Lees M., Staneley G.H.S. (1957). A simple method for the isolation and purification and total lipids from animal tissues. J. Biol. Chem..

[18] David F., Sandra P., Wylie P.L. (2003). Food Application: Improving the Analysis of Fatty Acid Methyl Esters Using Retention Time Locked Methods and Retention Time Databases.

[19] SPSS (2011). PASW Statistics 21.

[20] Bertol T.M., Oliveira E.A., Coldebella A., Kawski V.L., Scandolera A.J., Warpechowski M.B. (2015). Meat quality and cut yield of pigs slaughtered over 100 kg live weight. Arq. Bras. Med. Vet. Zootec..

[21] Cisneros F., Ellis M., McKeith F.K., McCaw J., Fernando R.L. (1996). Influence of slaughter weight on growth and carcass characteristics, commercial cutting and curing yields, and meat quality of barrows and gilts from two genotypes. J. Anim. Sci..

[22] Correa J.A., Faucitano L., Laforest J.P., Rivest J., Marcoux M., Gariépy C. (2006). Effects of slaughter weight on carcass composition and meat quality in pigs of two different growth rates. Meat Sci..

[23] Latorre M.A., Lázaro R., Valencia D.G., Medel P., Mateos G.G. (2004). The effects of gender and slaughter weight on growth performance, carcass traits, and meat quality characteristics of heavy pigs. J. Anim. Sci..

[24] Mesires N.T., Doumit M.E. (2002). Satellite cell proliferation and differentiation during postnatal growth of porcine skeletal muscle. Am. J. Physiol. Cell Physiol..

[25] Choi Y.M., Nam K.W., Choe J.H., Ryu Y.C., Wick M.P., Lee K., Kim B.C. (2013). Growth, carcass, fiber type, and meat quality characteristics in Large White pigs with different live weights. Livest. Sci..

[26] Choi Y.M., Oh H.K. (2016). Carcass performance, muscle fiber, meat quality, and sensory quality characteristics of crossbred pigs with different live weights. Korean J. Food Sci. An..

[27] Lee S.H., Kim J.M., Ryu Y.C., Ko K.S. (2016). Effects of morphological characteristics of muscle fibers on porcine growth performance and pork quality. Korean J. Food Sci. Anim. Resour..

[28] MFDS (2019). Standards for Labelling and Classification of Beef and Pork Cuts.

[29] NIAS (2021). Pork Carcass Yield Standards.

[30] Cava R., Estévez M., Ruiz J., Morcuende D. (2003). Physicochemical characteristics of three muscles from free-range reared Iberian pigs slaughtered at 90 kg live weight. Meat Sci..

[31] Realini C.E., Vénien A., Gou P., Gatellier P., Pérez-Juan M., Danon J., Astruc T. (2013). Characterization of *Longissimus thoracis*, *Semitendinosus* and *Masseter* muscles and relationships with technological quality in pigs. 1. Microscopic analysis of muscles. Meat Sci..

[32] Alonso V., Muela E., Gutiérrez B., Calanche J.B., Roncalés P., Beltrán J.A. (2015). The inclusion of Duroc breed in maternal line affects pork quality and fatty acid profile. Meat Sci..

[33] Razmaitè V., Juška R., Leikus R., Jatkauskienè V. (2021). Pork quality of two Lithuanian breeds: Effects of breed, gender and feeding regimen. Animals.

[34] Sarmeinto-García A., Vieira-Aller C. (2023). Improving fatty acid profile in native breed pigs using dietary strategies: A review. Animals.

[35] Li X., Hastie M., Waner R.D., Hewitt R.J.E., D’Souza D.N., Viejo C.G., Fuentes S., Ha M., Dunshea F.R. (2024). Consumer eating quality and physicochemical traits of pork *Longissimus* and *Semimembranosus* differed between genetic lines. Meat Sci..

[36] Listrat A., Lebret B., Louveau I., Astruc T., Bonnet M., Lefaucheur L., Picard B., Bugeon J. (2016). How muscle structure and composition influence meat and flesh quality. Sci. World J..

[37] Clausen I., Ovesen L. (2001). Proximate contents, losses and gains of fat, protein and water comparing raw, hospital- and household-cooked pork cuts. J. Food Comp. Anal..

[38] Kim J.H., Seong P.N., Jo S.H., Park B.Y., Hah K.H., Yu L.H., Lim D.G., Hwang I.H., Kim D.H., Lee J.M. (2008). Characterization of nutritional value for twenty-one pork muscles. Asian-Aust. J. Anim. Sci..

[39] Park J., Song S., Cheng H., Im C., Jung E.Y., Moon S.S., Choi J., Hur S.J., Joo S.T., Kim G.D. (2022). Comparison of meat quality and muscle fiber characteristics between porcine skeletal muscles with different architectures. Food Sci. Anim. Resour..

[40] Lawrie R.A., Leward D.A. (2006). Lawrie’s Meat Science.

[41] Thomas R., Anjaneyulu A.S.R., Kondaiah N. (2006). Quality and shelf life evaluation of emulsion and restructured buffalo meat nuggets at cold storage (4 ± 1 °C). Meat Sci..

[42] Jiménez-Colmenero F., Triki M., Herrero A.M., Rodríguez-Salas L., Ruiz-Capillas C. (2013). Healthy oil combination stabilized in a konjac matrix as pork fat replacement in low-fat, PUFA-enriched, dry fermented sausages. LWT–Food Sci. Technol..

[43] Salcedo-Sandoval L., Cofrades S., Ruiz-Capillas C., Jiménez-Colmenero F. (2014). Effect of cooking method on the fatty acid content of reduced-fat and PUFA-enriched pork patties formulated with a konjac-based oil bulking system. Meat Sci..

[44] Oshima I., Iwamoto H., Nakamura Y.N., Takayama K., Ono Y., Murakami T., Shiba N., Tabata S., Nishimura S. (2009). Comparative study of the histochemical properties, collagen content and architecture of the skeletal muscles of wild boar crossbred pigs and commercial hybrid pigs. Meat Sci..

[45] Lebret B., Ecolan P., Bonhomme N., Méteau K., Prunier A. (2015). Influence of production system in local and conventional pig breeds on stress indicators at slaughter, muscle and meat traits and pork eating quality. Animal.

[46] Nishimura T., Hattori A., Takahashi K. (1999). Structural changes in intramuscular connective tissue during the fattening of Japanese black cattle: Effect of marbling on beef tenderization. J. Anim. Sci..

[47] Karunaratne J.K., Ashton C.J., Stickland N.C. (2005). Fetal programming of fat and collagen in porcine skeletal muscles. J. Anat..

[48] Bailey A.J., Light N.D. (1989). Connective Tissue in Meat and Meat Products.

[49] Purslow P.P. (2005). Intramuscular connective tissue and its role in meat quality. Meat Sci..

[50] Jankowiak H., Cebulska A., Bocian M. (2021). The relationship between acidification (pH) and meat quality traits of polish white breed pigs. Eur. Food Res. Technol..

[51] Scheffler T.L., Gerrard D.E. (2007). Mechanisms controlling pork quality development: The biochemistry controlling postmortem energy metabolism. Meat Sci..

[52] England E.M., Matarneh S.K., Oliver E.M., Apaoblaza A., Scheffler T.L., Shi H., Gerrard D.E. (2016). Excess glycogen does not resolve high ultimate pH of oxidative muscle. Meat Sci..

[53] Huff-Lonergan E., Lonergan S.M. (2005). Mechanisms of water-holding capacity of meat: The role of postmortem biochemical and structural changes. Meat Sci..

[54] Fischer K. (2007). Drip loss in pork: Influencing factors and relation to further meat quality traits. J. Anim. Breed. Genet..

[55] Hoa V.B., Seol K.H., Seo H.W., Seong P.N., Kang S.M., Kim Y.S., Moon S.S., Kim J.H., Cho S.H. (2021). Meat quality characteristics of pork bellies in relation to fat level. Anim. Biosci..

[56] Jo K., Lee S., Kim H.B., Seong P.N., Jung S., Lee D.H. (2025). Cooking loss estimation of *semispinalis capitis* muscle of pork butt using a deep neural network on hyperspectral data. Meat Sci..

[57] Aaslyng M.D., Bejerholm C., Ertbjerg P., Bertram H.C., Andersen H.J. (2003). Cooking loss and juiciness of pork in relation to raw meat quality and cooking procedure. Food. Qual. Prefer..

[58] Tornberg E.V.A. (2005). Effects of heat on meat proteins—Implications on structure and quality of meat products. Meat Sci..

[59] Suman S.P., Joseph P. (2013). Myoglobin chemistry and meat color. Annu. Rev. Food Sci. Technol..

[60] Han J., Wang Y., Wang Y., Hao S., Zhang K., Tian J., Jin Y. (2024). Effect of changes in the structure of myoglobin on the color of meat products. Food Mater. Res..

[61] Hoa V.B., Cho S.H., Seong P.N., Kang S.M., Kim Y.S., Moon S.S., Choi Y.M., Kim J.H., Seol K.H. (2021). The significant influences of pH, temperature and fatty acids on meat myoglobin oxidation: A model study. J. Food Sci. Technol..

[62] Utrilla M.C., Soriano A., Ruiz A.G. (2010). Quality attributes of pork loin with different levels of marbling from Duroc and Iberian cross. J. Food Qual..

[63] Tapp W.N., Yancey J.W.S., Apple J.K. (2011). How is the instrumental color of meat measured?. Meat Sci..

[64] Choi Y.S., Lee J.K., Jung J.T., Jung Y.C., Jung J.H., Jung M.O., Choi Y.I., Jin S.K., Choi J.S. (2016). Comparison of meat quality and fatty acid composition of longissimus muscles from purebred pigs and three-way crossbred LYD pigs. Korean J. Food Sci. Anim. Resour..

[65] Wood J.D., Enser M., Fisher A.V., Nute G.R., Sheard P.R., Richardson R.I., Hughes S.I., Whittington F.M. (2008). Fat deposition, fatty acid composition and meat quality: A review. Meat Sci..

[66] Realini C.E., Pérez-Juan M., Gou P., Díaz I., Sárraga C., Gatellier P., García-Regueiro J.A. (2013). Characterization of Longissimus thoracis, Semitendinosus and Masseter muscles and relationships with technological quality in pigs. 2. Composition of muscles. Meat Sci..

